# Waterbirth: a national retrospective cohort study of factors associated with its use among women in England

**DOI:** 10.1186/s12884-021-03724-6

**Published:** 2021-03-26

**Authors:** H. Aughey, J. Jardine, N. Moitt, K. Fearon, J. Hawdon, D. Pasupathy, I. Urganci, NMPA Project Team, T. Harris

**Affiliations:** 1 National Maternity and Perinatal Audit (NMPA), RCOG Centre for Quality Improvement and Clinical Audit, Royal College of Obstetricians and Gynaecologists, 10 –18 Union Street, London, SE1 1SZ UK; 2 University Hospitals Bristol NHS Foundation Trust, Bristol, UK; 3 Department of Health Service Research and Policy, London School of Hygiene and Tropical Medicine, London, UK; 4 Population Health Analytics, Cerner, London, UK; 5 Centre for Reproduction Research, De Montfort University, Leicester, UK; 6 Royal Free London NHS foundation Trust, London, UK; 7 Specialty of Obstetrics, Gynaecology and Neonatology, Westmead Clinical School, University of Sydney, Sydney, Australia; 8 Faculty of Health and Life Sciences, De Montfort University, Leicester, UK

**Keywords:** Waterbirth, Childbirth, Labour care

## Abstract

**Background:**

Waterbirth is widely available in English maternity settings for women who are not at increased risk of complications during labour. Immersion in water during labour is associated with a number of maternal benefits. However for birth in water the situation is less clear, with conclusive evidence on safety lacking and little known about the characteristics of women who give birth in water.

This retrospective cohort study uses electronic data routinely collected in the course of maternity care in England in 2015–16 to describe the proportion of births recorded as having occurred in water, the characteristics of women who experienced waterbirth and the odds of key maternal and neonatal complications associated with giving birth in water.

**Methods:**

Data were obtained from three population level electronic datasets linked together for the purposes of a national audit of maternity care. The study cohort included women who had no risk factors requiring them to give birth in an obstetric unit according to national guidelines. Multivariate logistic regression models were used to examine maternal (postpartum haemorrhage of 1500mls or more, obstetric anal sphincter injury (OASI)) and neonatal (Apgar score less than 7, neonatal unit admission) outcomes associated with waterbirth.

**Results:**

46,088 low and intermediate risk singleton term spontaneous vaginal births in 35 NHS Trusts in England were included in the analysis cohort. Of these 6264 (13.6%) were recorded as having occurred in water. Waterbirth was more likely in older women up to the age of 40 (adjusted odds ratio (adjOR) for age group 35–39 1.27, 95% confidence interval (1.15,1.41)) and less common in women under 25 (adjOR 18–24 0.76 (0.70, 0.82)), those of higher parity (parity ≥3 adjOR 0.56 (0.47,0.66)) or who were obese (BMI 30–34.9 adjOR 0.77 (0.70,0.85)). Waterbirth was also less likely in black (adjOR 0.42 (0.36, 0.51)) and Asian (adjOR 0.26 (0.23,0.30)) women and in those from areas of increased socioeconomic deprivation (most affluent versus least affluent areas adjOR 0.47 (0.43, 0.52)).

There was no association between delivery in water and low Apgar score (adjOR 0.95 (0.66,1.36)) or incidence of OASI (adjOR 1.00 (0.86,1.16)). There was an association between waterbirth and reduced incidence of postpartum haemorrhage (adjOR 0.68 (0.51,0.90)) and neonatal unit admission (adjOR 0.65 (0.53,0.78)).

**Conclusions:**

In this large observational cohort study, there was no association between waterbirth and specific adverse outcomes for either the mother or the baby. There was evidence that white women from higher socioeconomic backgrounds were more likely to be recorded as giving birth in water. Maternity services should focus on ensuring equitable access to waterbirth.

**Supplementary Information:**

The online version contains supplementary material available at 10.1186/s12884-021-03724-6.

## Background

The advantages of immersion in water during the first stage of labour are well described in the literature. These include a decreased requirement for analgesia in labour, decreased obstetric interventions in labour and an increased sense of maternal satisfaction with and control over labour [1–6]. However, immersion in water during labour must be differentiated from ‘waterbirth’, defined as the birth of the baby under water [1, 7]. There exists a growing body of observational data which generally supports the safety of birth in water for both the mother and the baby, [1, 3, 8–13] including a number of recent meta-analyses which do not show evidence of harm to the neonate associated with birth in water for low-risk women [11–13]. Nonetheless, the most recently revised Cochrane review (updated in 2018) concludes that, for immersion during the second stage of labour there is no evidence of harm but that the evidence is limited [7].

The lack of conclusive evidence regarding waterbirth is reflected in UK guidance provided by the National Institute of Health and Care Excellence (NICE), which states that women should be advised that ‘there is insufficient high-quality evidence to either support or discourage giving birth in water’ [14]. Concerns have been raised that there may be associated increased risks: for the baby, respiratory difficulties and umbilical cord avulsion; and for the mother, perineal tearing [9, 10, 15–19]. Randomised trials in labour present challenges related to willingness to be randomised, [20] the nature of the intervention and the size of trial required [1, 7]. Therefore, large observational studies remain of use in adding to the body of evidence surrounding waterbirth [7, 21].

Many women giving birth in England are offered the choice of labouring in and/or giving birth in water. The practice is widely available throughout English maternity settings, with guidance from professional bodies supporting its use [14, 22] and birthing pools available in the majority of settings [23]. CQC survey data suggests that 11% of women who delivered vaginally did so in water and that this proportion has increased steadily since 2007 [24]. Beyond this, little is known about the incidence of waterbirth in England, nor about the characteristics of women who give birth in water [2].

The aims of this study were to describe the proportion of births in England during the financial year 2015/16 that were recorded as having occurred in water; the characteristics of women recorded as giving birth in water; and associated defined maternal and neonatal outcomes.

## Methods

This was a retrospective cohort study using data routinely collected for maternity care in the English National Health Service between 1st April 2015 and 31st March 2016.

The data for this study were obtained from three population-level electronic datasets, linked together for the purposes of a national audit of maternity care. Data were obtained from maternity data extracts from hospital trust Maternity Information Systems (MISs) in England, as described elsewhere [25]. Data obtained from MISs includes information relevant to the birth episode and the outcomes thereof. Hospital data were obtained from Hospital Episode Statistics (HES), an administrative dataset that is primarily used for provider payment. HES contains diagnostic and procedure codes allowing for longitudinal follow up of the women included in the cohort. Linkage between MISs and HES was performed by a trusted third party (NHS Digital) using a deterministic algorithm based on the mother and baby’s dates of birth, NHS numbers and maternal postcode. This process achieved 92% case ascertainment when compared with total registrable births reported by the Office for National Statistics (ONS) for the same time period [25]. Neonatal admission data were obtained from the National Neonatal Research Dataset and linked as described elsewhere [26].

Information was available about maternal characteristics: age, ethnicity, body mass index at first pregnancy appointment, parity, previous caesarean section, and current and previously recorded diagnostic codes. Socio-economic status was derived from the government index of multiple deprivation and based on the woman’s local area of approximately 1500 residents, derived from the postcode in her maternity record [27]. Neonatal characteristics included gestation, mode of delivery, birthweight, and the baby’s condition at birth. The exposure of interest, waterbirth, was coded in the MIS field ‘waterdelivery’ as yes, no or missing. The data specification indicated that the field corresponded to whether the birth occurred in water [28]. A full list of the variables used and the original datasets they were derived from is available in Supplementary Table 1.

For maternal and neonatal outcomes, information was available about complications including obstetric anal sphincter injury (OASI), postpartum haemorrhage (PPH), Apgar scores and whether the baby required admission to neonatal care [25, 26].

35 trusts in England (of 134 providing maternity care) [23] had sufficiently high completeness of information about waterbirth in their maternity information system records to be included in the study (details of data sources and criteria used are available in supplementary information Table 1). The cohort included women giving birth to live-born singleton babies in these 35 trusts during the financial year 2015–16 who had sufficiently complete data relating to the exposures and outcomes of interest in their linked MIS-HES record. The study population was then defined as women who were eligible for waterbirth: aged between 18 and 44, with a BMI of less than 35, and without pre-existing medical conditions, previous obstetric complication or conditions arising in pregnancy that would make them ‘high risk’ according to the UK NICE Guidelines on Intrapartum Care [14, 29] (Supplementary Table 2). This cohort was further restricted to women who gave birth at term (37^+ 0^ to 42^+ 6^ weeks gestation) and had a spontaneous vaginal delivery; breech, instrumental and caesarean births were excluded.

We present descriptive statistics of the characteristics of women in the cohort and the rate of waterbirth by individual characteristics. Continuous variables were categorised: age was grouped in five year increments, BMI into World Health Organisation categories, [30] and birthweight into less than 2500 g, 2500 g–3999 g and 4000 g or more. Parity of 3 or more was treated as a single category.

Univariate and multivariate logistic regression models were used to compare characteristics of women who gave birth in water. Multivariate logistic regression models were used to examine maternal (PPH ≥1500 ml, OASI) and neonatal outcomes (Apgar score < 7 at five minutes, neonatal unit admission) associated with delivery in water. Adjustment was made for maternal BMI, ethnicity, socio-economic deprivation, parity, and birthweight.

In order to evaluate whether the observed difference was due to heterogeneity in the group of women examined that could not be captured by the available data (unmeasured confounding) or treatment in labour such as the use of augmentation, a sensitivity analysis was planned to restrict to women who gave birth in a midwife-led setting only (recorded place of birth of either home or a midwife-led unit). In the UK women are eligible to give birth in a midwife-led setting if they have no additional risks in pregnancy, and do not require medical intervention during labour.

To understand the generalisability of results, distribution of trust size and location was compared between included and excluded trusts using chi squared tests.

## Results

**Fig. 1:**
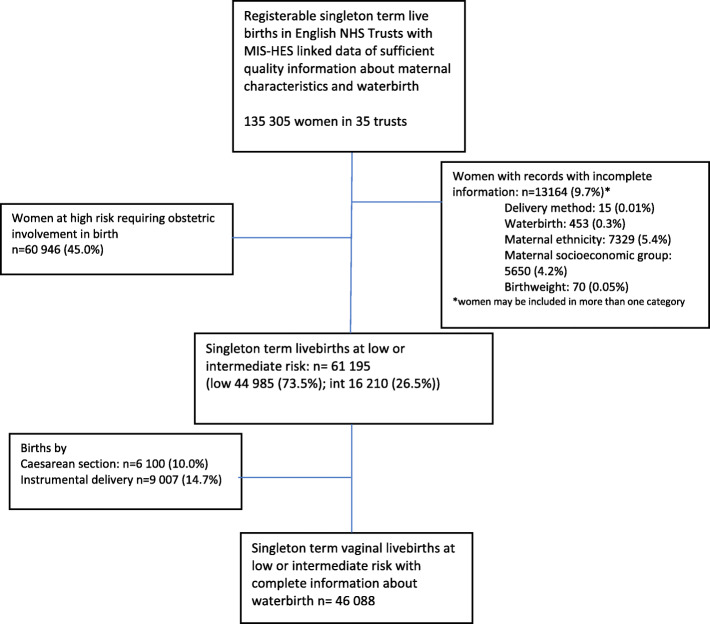
Flowchart of inclusion in study

127,398 records with adequate data completeness from 35 English NHS trusts were identified. After the application of exclusions, 46,088 singleton term spontaneous vaginal births to low and intermediate risk women were included in the analysis cohort (Fig. 1, Table 1). Of these, 6264 (13.6%) were recorded as having occurred in water (Table 1). Information about whether the baby was admitted to neonatal care was available for 41,596 women.

**Table 1:** Rate of waterbirth according to maternal and obstetric risk factors

Characteristic	Number of women in group	Number of women who give birth in water	Rate of birth in water (%)	Crude OR (95% CI)	Adjusted OR (95% CI)	p (Wald test)
All women	46,088	6264	13.6			
Age						
18–24	9569	993	10.4	0.78 (0.72,0.85)	0.76 (0.70,0.82)	< 0.001
25–29	13,810	1783	12.9	Ref	Ref	
30–34	14,359	2281	15.9	1.27 (1.19,1.36)	1.17 (1.09,1.25)	
35–39	7142	1103	15.4	1.23 (1.14,1.34)	1.12 (1.03,1.22)	
40–44	1208	104	8.6	0.64 (0.52,0.78)	0.60 (0.48,0.74)	
Maternal BMI						
18.5–24.9	27,011	3890	14.4	Ref	Ref	< 0.001
25.0–29.9	13,698	1818	13.3	0.91 (0.86,0.97)	0.98 (0.92,1.04)	
30–34.9	5379	556	10.3	0.69 (0.62,0.75)	0.77 (0.70,0.85)	
Parity						
0	17,206	2393	13.9	Ref	Ref	
1	18,914	2835	15.0	1.09 (1.03,1.16)	1.01 (0.95,1.08)	
2	6691	798	11.9	0.84 (0.77,0.91)	0.86 (0.79,0.94)	
3+	3277	238	7.3	0.48 (0.42,0.56)	0.56 (0.47,0.66)	
Ethnicity						
White	37,581	5793	15.4	Ref	Ref	< 0.001
Black	2339	139	5.9	0.35 (0.29,0.41)	0.42 (0.35, 0.50)	
Asian	4506	189	4.2	0.24 (0.21,0.28)	0.26 (0.23,0.30)	
Other	143	143	8.6	0.52 (0.43,0.61)	0.56 (0.47,0.66)	
Socioeconomic status						
Least deprived = 1	9450	1787	18.9	Ref	Ref	< 0.001
2	7278	1180	16.2	0.83 (0.77,0.90)	0.89 (0.82, 0.96)	
3	10,120	1448	14.3	0.72 (0.66, 0.77)	0.81 (0.75, 0.87)	
4	10,049	1140	11.3	0.55 (0.51,0.59)	0.68 (0.62, 0.74)	
Most deprived = 5	9191	709	7.7	0.36 (0.33,0.39)	0.47 (0.43, 0.52)	
Birthweight						
< 2500 g	594	15	2.5	0.17 (0.10,0.28)	0.19 (0.11, 0.32)	< 0.001
2500-4000 g	40,204	5385	13.4	Ref	Ref	
> 4000 g	5290	864	16.3	1.26 (1.17,1.37)	1.12 (1.03,1.21)	

This table shows the rate of waterbirth in 46,088 women who had an unassisted vaginal birth in England in 2015/16 according to maternal and obstetric risk factors

In this cohort of women without pre-existing comorbidities or obstetric risk factors, waterbirth was more likely in women aged 30–34 (15.9%, adjusted odds ratio (adjOR) 1.17 (95% confidence interval 1.09, 1.25)) and 35–39 (15.4%, adjOR 1.12 (1.03,1.22)) compared to those aged 25–29 (12.9%). Waterbirth was less common in women over the age of 40 (8.6%, adjOR 0.60 (0.48,0.74)) and in younger women, with only 10.4% of those age 18–24 recorded as having a waterbirth (adjOR 0.76 (0.70, 0.82)) (Table 1).

Waterbirth was less common in women who were obese (BMI 30–34.9 10.3%, adjOR 0.77 (0.70,0.85)) and was less common in women with a parity of 3 or more (7.3%, adjOR 0.56 (0.47,0.66)) (Table 1).

The strongest associations were seen with ethnic group and deprivation. Waterbirth was less likely in women of Black (5.9%, adjOR 0.42 (0.36, 0.51)) or Asian (4.2%, adjOR 0.26 (0.23,0.30)) ethnicity. Waterbirth became less frequent with increasing socio-economic deprivation, with women in the most deprived quintile of areas only half as likely to have a waterbirth as those in the most affluent areas (7.7% compared to 18.9%, adjOR 0.47 (0.43, 0.52)) (Table 1).

There was no association between birth in water and low Apgar score (adjOR 0.95 (0.66,1.36)) or OASI (adjOR 1.00 (0.86,1.16). There was an association between waterbirth and reduced admission to a neonatal unit (adjOR 0.65 (0.53,0.78)) and an association with reduced PPH > = 1500mls (adjOR 0.68 (0.51,0.90)) (Table 2). These associations were unchanged in a sensitivity analysis which restricted the cohort to only those with their place of birth recorded as a midwife-led unit (Supplementary Table 3).

**Table 2:** Rates of complications among 46,088 women who had a spontaneous vaginal delivery in 2015–16

	Number of women experiencing outcome (%)			Crude OR (95% CI)	Adjusted^a^ OR (95% CI)	*p* value
	among all women (***n*** = 46,088)	among women recorded as not having waterbirth (***n*** = 39,824)	among women recorded as having waterbirth (***n*** = 6284)			
Maternal						
Obstetric anal sphincter injury	1480 (3.21%)	1259 (3.16%)	221 (3.53%)	1.12 (0.97, 1.30)	1.00 (0.86,1.16)	0.99
Postpartum haemorrhage > = 1500 ml	552 (1.20%)	496 (1.25%)	56 (0.89%)	0.72 (0.54,0.94)	0.68 (0.51,0.90)	0.007
Neonatal						
Apgar< 7 at 5 min of age	270 (0.59%)	234 (0.59%)	36 (0.57%)	0.98 (0.69,1.39)	0.95 (0.66,1.36)	0.78
Neonatal admission^b^	1287 (3.09%)	1168 (2.93%)	119 (3.25%)	0.64 (0.53,0.78)	0.65 (0.53,0.78)	< 0.001

^a^adjusted for factors described in Table 1

^b^ in a restricted cohort of 41,596 women for whom information about neonatal admission was available

A comparison of characteristics between trusts that were and were not included in the study demonstrated that included trusts were broadly representative of England as a whole in terms of size and location (Supplementary Table 4).

## Discussion

### Summary of results

This study found that in this restricted cohort of women giving birth in England, 13.6% were recorded as having a waterbirth. Women of ethnic minority origin, younger women and women of more deprived socioeconomic status are less likely to give birth in water. For the mother, waterbirth was associated with reduced PPH and no association was shown between waterbirth and OASI. For the baby, there was no association between waterbirth and low Apgar score, and neonatal unit admission was less likely in the group born in water. This study therefore shows no association between waterbirth and these adverse outcomes for mother or baby.

### Comparison with other studies

This study found that increasing age, with the exception of women over 40 years old, is associated with increasing likelihood of waterbirth. This association is consistent with a previous study finding that women under 25 are less likely to use water for analgesia during labour [8, 31].

In this cohort, socioeconomic deprivation was strongly associated with decreasing likelihood of giving birth in water. Women living in the most deprived postcode areas were less than half as likely to give birth in water as those in the least deprived group. An existing study similarly found that socioeconomically deprived women are less likely to labour in water [31]. In our cohort, women of Black and Asian ethnic origin were substantially less likely to have a waterbirth than white women. This persisted even after adjustment for other factors such as birthweight and obesity which are associated with ethnicity. Recent studies from the USA also report ethnic differences between women who labour or give birth in water and women who do not [6, 9].

Although it is possible that the reason for this discrepancy is that women in these groups laboured in water but left the pool prior to delivery, it is probable that these disparities reflect inequitable access to birthing pools in England. It may be that some groups of women are not aware that waterbirth is an option available to them, or that some groups are less empowered and therefore less able to advocate for their own preferences during labour and birth. This is supported by a secondary analysis of the Birthplace cohort study which found that it is not only women experiencing socioeconomic deprivation who are less likely to labour in water, but also those who do not speak fluent English and those who are unsupported by a partner [31].

Obese women were found to be less likely to give birth in water. This finding is consistent with existing UK guidelines where women with a BMI of between 30 and 35 are not always offered care in a midwife-led setting [14]. It has elsewhere been shown that, among women who intend to give birth in water, multiparous women are more likely to do so [32]. Our study, in which the group who did not give birth in water includes all low-risk women who had a spontaneous vaginal birth, showed that women of higher parity are less likely to deliver in water. This may reflect individual preferences, lower request for analgesia or reduced time available to allow for waterbirth to occur.

Whilst some previous studies identify birth in water as an independent risk factor for OASI [16, 33] and others suggest an association with an increased risk of more minor genital tract tears, [1, 33, 34] the majority of the published literature reports either no association or a decreased incidence of severe perineal tears and episiotomy associated with birth in water [1, 6, 8, 9, 35–37]. In agreement with this, our study found no evidence of increased incidence of OASI following waterbirth.

Waterbirth in this cohort was associated with a reduced risk of a PPH of 1500 ml or more. This is to be expected, since a number of existing studies have also found waterbirth to be equivalent if not superior to birth not in water in terms of association with PPH [1, 35, 38]. However, this finding must be treated with some caution; although the finding was robust to a sensitivity analysis where the cohort was restricted to births that occurred in a midwife led setting and therefore without augmentation, we were unable to control for slow labour progress which is associated with PPH. Furthermore, there may be bias in the recording of PPH of 1500 ml or more, as quantifying blood loss in a birthing pool may be challenging. It is a strength of this study that we were able to adjust for parity and birthweight, both factors associated with OASI and PPH [39–41].

Although the possibility of rare but serious adverse neonatal outcomes remains, the conclusion may be drawn from the existing published evidence that, for most neonatal outcomes, there is no evidence of any significant differences between birth in water and birth not in water [9, 11–13, 34, 37]. This large study found no association between birth in water and low Apgar scores and also that babies born underwater were less likely to be admitted to a neonatal unit, thereby adding to the body of evidence supporting the safety for the baby of delivery in water.

### Strengths and limitations

To our knowledge, this is the largest published study of births in water in the UK. Furthermore, since this study makes use of routinely collected data from an unselected population, the risk of selection bias is reduced. Included trusts were broadly representative in size and location of trusts throughout England. These results are, therefore, readily generalisable to the population in England.

For maternal and neonatal outcomes other than PPH, there is no evidence of a statistically significant association between water birth and adverse outcome. It is possible that this is because this study, although relatively large, is not large enough to detect smaller differences in adverse outcomes in women giving birth in water.

The central limitation is data quality and completeness. Data were only available for a minority of trusts in England; in others, the proportion of women for whom the field ‘waterdelivery’ was empty was too high to draw conclusions. Furthermore, no information was available about the women who labour in water but do not deliver in water. There is also uncertainty around the quality of recording of routine data relating to birth in water. We were not able to access paper clinical records and therefore no validation of this electronic data field has taken place. It is possible that a proportion of women who are recorded as having had a waterbirth may have spent time in a pool during labour, but not actually given birth in water.

This is an observational study and thus no conclusions about causation can be inferred from these results. Furthermore, there are likely to be unmeasured confounding factors that cannot be captured in this routinely collected dataset. This study attempts to limit these by restricting the cohort to women without risk factors requiring birth on an obstetric unit who gave birth by normal vaginal delivery and additionally through a sensitivity analysis including only women who gave birth in a midwife-led setting. However, even within this restricted cohort, it is likely that unmeasurable differences remain between women who delivered in water and those who did not.

## Conclusions

This large observational cohort study shows that, in this cohort of women without risk factors that would prompt the recommendation of birth in an obstetric unit, there was no association between waterbirth and the specific adverse maternal or neonatal outcomes investigated. This study therefore adds to the body of evidence that is available to support women in making decisions and healthcare professionals in offering advice about giving birth in water. There remains a need for further research to consider the safety and benefits of waterbirth for women with specific risk factors, such as those with an elevated BMI.

A key finding of this study is that women from socioeconomically deprived backgrounds and ethnic minorities are less likely to give birth in water. Crucially, this raises the possibility that access to waterbirth is not equally distributed between socioeconomic and ethnic groups in England. We suggest that qualitative research to explore the experiences of women from different ethnic and socioeconomic backgrounds should be a priority for future waterbirth research. Furthermore, healthcare providers should ensure that these groups of women are given relevant information and are empowered to make choices about where and how they give birth, including the use of waterbirth.

Improved recording of waterbirth in electronic datasets, both in terms of improved completeness of existing variables and the inclusion of additional information relating to the use of water in labour, will assist with future understanding of the epidemiology and associated risks of giving birth in water.

## Supplementary Information

**Additional file 1: Supplementary Information 1**. Sources of information used in the data set and criteria for inclusion. A full list of the variables used and the original datasets they were derived from as well as the quality criteria required for inclusion.

**Additional file 2: Supplementary Information 2**. Table: Summary of NICE Guideline CG90: Intrapartum care for low risk pregnancies. A table summarising the NICE Guideline CG90 defining the characteristics associated with low, medium and high-risk pregnancies.

**Additional file 3: Supplementary Information 3**. Table. Sensitivity Analysis: Rates of complications of waterbirth in 30,993 low-risk women who gave birth in a midwife-led unit. A table displaying the results of a sensitivity analysis with a cohort restricted to women giving birth in a midwife lead setting.

**Additional file 4: Supplementary Information 4**. Table. Characteristics of included and excluded trusts. A table comparing size and geographical regions of included and excluded trusts.

## Data Availability

The data that support the findings of this study are held by NHS Digital (HES) and the RCOG (MIS) but restrictions apply to the availability of these data, which were used under license for the current study, and so are not publicly available. Data are however available from the authors upon reasonable request and with permission of NHS Digital and HQIP.

## Abbreviations

NMPA: National Maternity and Perinatal Audit HES Hospital Episode Statistics
PPH: Post-partum haemorrhage
OASI: Obstetric anal sphincter injury
MIS: Maternity Information System
NNRD: National Neonatal Research Database
RCOG: Royal College of Obstetricians and Gynaecologists
HQIP: Healthcare Quality Improvement Partnership
BMI: Body Mass Index
NICE: National Institute for Health and Care Excellence
