# Correction to: Waterbirth: a national retrospective cohort study of factors associated with its use among women in England

**DOI:** 10.1186/s12884-021-03871-w

**Published:** 2021-05-19

**Authors:** H. Aughey, J. Jardine, N. Moitt, K. Fearon, J. Hawdon, D. Pasupathy, I. Urganci, NMPA Project Team, T. Harris

**Affiliations:** 1 National Maternity and Perinatal Audit (NMPA), RCOG Centre for Quality Improvement and Clinical Audit, Royal College of Obstetricians and Gynaecologists, 10 –18 Union Street, London, SE1 1SZ UK; 2 University Hospitals Bristol NHS Foundation Trust, Bristol, UK; 3 Department of Health Service Research and Policy, London School of Hygiene and Tropical Medicine, London, UK; 4 Population Health Analytics, Cerner, London, UK; 5 Centre for Reproduction Research, De Montfort University, Leicester, UK; 6 Royal Free London NHS foundation Trust, London, UK; 7 Specialty of Obstetrics, Gynaecology and Neonatology, Westmead Clinical School, University of Sydney, Sydney, Australia; 8 Faculty of Health and Life Sciences, De Montfort University, Leicester, UK

**Correction to: BMC Pregnancy Childbirth 21, 256 (2021)**

**https://doi.org/10.1186/s12884-021-03724-6**

Following publication of the original article [1], the authors reported an error to the last row of Table 2.

The table should read:

**Table:** 

	Number of women experiencing outcome (%)			Crude OR (95% CI)	Adjusted^**a**^ OR (95% CI)	***p*** value
	among all women	among women recorded as not having waterbirth	among women recorded as having waterbirth			
Neonatal admission^b^	1287 (3.09%)	1168 (3.25%)	119 (2.11%)	0.64 (0.53,0.78)	0.65 (0.53,0.78)	<0.001

The original article [1] has been updated.
