# Supplementary material for: Waterbirth: a national retrospective cohort study of factors associated with its use among women in England
Source: BMC Pregnancy Childbirth. 2021 Mar 26;21:256. doi: 10.1186/s12884-021-03724-6 (PMC8004456; doi:10.1186/s12884-021-03724-6)
Supplement: Supplementary file 1 — Additional file 1: Supplementary Information 1. Sources of information used in the data set and criteria for inclusion. A full list of the variables used and the original datasets they were derived from as well as the quality criteria required for inclusion. [file 12884_2021_3724_MOESM1_ESM.docx]

##### Supplementary Information 1. Sources of information used in the data set and criteria for inclusion

|  | Primary source of information (secondary if missing in primary source) | Quality criteria associated with inclusion |
| --- | --- | --- |
| **Maternal characteristics** |  |  |
| Age | MIS | Record contains complete information about maternal age |
| BMI | MIS | Trust has >70% records complete for BMI; Record contains complete information about maternal BMI |
| Ethnicity | MIS (HES) | Record contains complete information |
| Parity | MIS (HES) | Record contains complete information |
| Previous obstetric complications | HES (MIS) | n/a |
| Maternal comorbidities | HES | n/a |
| Socioeconomic status | MIS | Record contains complete information |
| Obstetric complications in this pregnancy | HES | n/a |
| **Birth characteristics** |  |  |
| Birthweight | MIS | Record contains complete information about |
| Delivery method | MIS/HES | Trust has >70% records complete; Record contains complete information |
| Birth in water | MIS | Trust has >70% records complete for both all records and for vaginal births; Record contains complete information |
| Singleton | MIS | Trust has >70% records complete; Record contains complete information |
| Gestational age | MIS/HES | Trust has >70% records complete; Record contains complete information |
| Live birth | MIS | Record contains complete information |
| Blood loss | MIS | Trust has >70% records complete; Record contains complete information |
| Apgar at 5 minutes of age | MIS | Trust has >70% records complete; Record contains complete information |
| Presence of obstetric anal sphincter injury | MIS/HES | Record contains complete information |
| Neonatal admission | NNRD | >=80% of records in neonatal unit for trust have a corresponding maternity record |
| Place of birth | MIS | Record contains complete information |
