# Supplementary material for: Waterbirth: a national retrospective cohort study of factors associated with its use among women in England
Source: BMC Pregnancy Childbirth. 2021 Mar 26;21:256. doi: 10.1186/s12884-021-03724-6 (PMC8004456; doi:10.1186/s12884-021-03724-6)
Supplement: Supplementary file 2 — Additional file 2: Supplementary Information 2. Table: Summary of NICE Guideline CG90: Intrapartum care for low risk pregnancies. A table summarising the NICE Guideline CG90 defining the characteristics associated with low, medium and high-risk pregnancies. [file 12884_2021_3724_MOESM2_ESM.docx]

##### Supplementary Information 2. Table: Summary of NICE Guideline CG90: Intrapartum care for low risk pregnancies

| Risk status | Low (birth recommended in a midwife-led setting) | Intermediate (discussion about choice of place of birth) | High  (birth recommended in obstetric setting) |
| --- | --- | --- | --- |
| BMI | <30kg/m^2^ | 30-34.99kg/m^2^ | ≥35kg/m^2^ |
| Previous medical history | Stable mild medical conditions (asthma, hypothyroidism) | Mild medical conditions or condition | Medical comorbidity (e.g. hypertension)  Previous surgery on the uterus e.g. myomectomy |
| Previous obstetric history | No significant history | Parity of 4 or more.  Previous events that are unlikely to occur again, for example stillbirth of known cause.  Previous mild complications not known to occur in this pregnancy, e.g. mild pre-eclampsia | Previous caesarean section  Previous events which may occur again, for example severe pre-eclampsia or stillbirth of unknown aetiology |
| Current obstetric complications | Nil |  | Conditions or suspected conditions in mother, such as pre-eclampsia  Fetal complications such as anomaly, multiple pregnancy or suspected macrosomia |
