# Supplementary material for: Waterbirth: a national retrospective cohort study of factors associated with its use among women in England
Source: BMC Pregnancy Childbirth. 2021 Mar 26;21:256. doi: 10.1186/s12884-021-03724-6 (PMC8004456; doi:10.1186/s12884-021-03724-6)
Supplement: Supplementary file 3 — Additional file 3: Supplementary Information 3. Table. Sensitivity Analysis: Rates of complications of waterbirth in 30,993 low-risk women who gave birth in a midwife-led unit. A table displaying the results of a sensitivity analysis with a cohort restricted to women giving birth in a midwife lead setting. [file 12884_2021_3724_MOESM3_ESM.docx]

##### Supplementary Information 3. Table. Sensitivity Analysis: Rates of complications of waterbirth in 30 993 low-risk women who gave birth in a midwife-led unit

|  | Overall number of women experiencing outcome (%) | Number experiencing outcome among women recorded as having waterbirth (%) | Crude OR (95% CI) | Adjusted* OR (95% CI) | p |  |
| --- | --- | --- | --- | --- | --- | --- |
| Maternal |  |  |  |  |  |  |
| Obstetric anal sphincter injury | 930 (3.01) | 193 (3.49) | 1.21 (1.02,1.42) | 1.09 (0.92,1.28) | 0.32 |  |
| Postpartum haemorrhage >=1500ml | 351 (1.13) | 48 (0.87) | 0.73 (0.53,0.98) | 0.69 (0.50,0.94) | 0.02 |  |
| Neonatal |  |  |  |  |  |  |
| Apgar<7 at 5 minutes of age | 172 (0.56) | 35 (0.63) | 1.17 (0.81,1.70) | 1.12 (0.77,1.63) | 0.56 |  |
| Neonatal admission** | 842 | 101 | 0.62 (0.50,0.77) | 0.62 (0.50,0.77) | <0.001 |  |
| *adjusted for factors described in table 1  ** in a restricted cohort of 27 958 women for whom information about neonatal admission was available. | | | | | | |
