# Supplementary material for: Waterbirth: a national retrospective cohort study of factors associated with its use among women in England
Source: BMC Pregnancy Childbirth. 2021 Mar 26;21:256. doi: 10.1186/s12884-021-03724-6 (PMC8004456; doi:10.1186/s12884-021-03724-6)
Supplement: Supplementary file 4 — Additional file 4: Supplementary Information 4. Table. Characteristics of included and excluded trusts. A table comparing size and geographical regions of included and excluded trusts. [file 12884_2021_3724_MOESM4_ESM.docx]

##### Supplementary Information 4. Table. Characteristics of included and excluded trusts

|  | Included trusts (n=35) | Excluded trusts (n=89) | P value* |
| --- | --- | --- | --- |
| **Size of trust (number of births)** |  |  |  |
| <2500 | 3 | 14 | 0.49 |
| 2500-3999 | 7 | 22 |  |
| 4000-5999 | 17 | 31 |  |
| >=6000 | 8 | 22 |  |
| **Region** |  |  |  |
| London | 5 | 12 | 0.09 |
| South East/Home Counties | 11 | 11 |  |
| East of England | 0 | 3 |  |
| Midlands | 5 | 17 |  |
| North East & Yorkshire | 3 | 18 |  |
| North West | 3 | 13 |  |
| South West | 7 | 9 |  |

| *using chi2 test of association |
| --- |
